# The Multivariate Effect of Ketamine on PTSD: Systematic Review and Meta-Analysis

**DOI:** 10.3389/fpsyt.2022.813103

**Published:** 2022-03-09

**Authors:** Rui Du, Ruili Han, Kun Niu, Jiaqiao Xu, Zihou Zhao, Guofang Lu, Yulong Shang

**Affiliations:** ^1^Institute for Biomedical Sciences of Pain, Tangdu Hospital, Fourth Military Medical University, Xi'an, China; ^2^Department of Anaesthesiology, Tangdu Hospital, Fourth Military Medical University, Xi'an, China; ^3^Department of Anaesthesiology, Key Laboratory of Carcinogenesis and Translational Research (Ministry of Education/Beijing), Peking University Cancer Hospital and Institute, Beijing, China; ^4^Tangdu Hospital, Fourth Military Medical University, Xi'an, China; ^5^Department of Physiology and Pathophysiology, National Key Discipline of Cell Biology, Fourth Military Medical University, Xi'an, China; ^6^State Key Laboratory of Cancer Biology and National Clinical Research Center for Digestive Diseases, Xijing Hospital of Digestive Diseases, Fourth Military Medical University, Xi'an, China

**Keywords:** ketamine, analgesia, meta-analysis, PTSD, chronic PTSD

## Abstract

**Background:**

Post-traumatic stress disorder (PTSD) is a serious stress-related disorder caused by traumatic experiences. However, identifying a key therapy that can be used for PTSD treatment remains difficult. Ketamine, a well-known dissociative anesthetic, is considered safe to be used in anesthesia, pain management, and antidepressant actions since 1970. At present, it is still controversial whether PTSD can be treated with ketamine. The authors performed a meta-analysis to determine whether the use of perioperative ketamine lowers the incidence of PTSD.

**Methods:**

Cochrane Central Register of Controlled Trials, Embase, PubMed, and Web of Science were searched to examine the use of ketamine for the treatment of PTSD among soldiers with combating experience. Studies were included if they were randomized placebo-controlled, case-control, and cohort studies. The primary outcome was the incidence of PTSD in the later stage of the wounded or burn soldiers. The secondary outcome was the influence of ketamine on PTSD-scale scores for early and chronic PTSD, respectively.

**Results:**

Our search yielded a total of three studies (*n* = 503 patients) comparing the use of ketamine (*n* = 349) to control (*n* = 154). The available evidence showed no significant difference in the incidence of PTSD between combatant soldiers on the battlefield with or without ketamine treatment (risk ratio = 0.81, 95% CI, 0.63–1.04; *P* = 0.10). In 65 patients from three trials, ketamine was not only ineffective in treating early PTSD but also lead to exacerbation of the disease (risk ratio = 2.45, 95% CI, 1.33–3.58; *P* < 0.001). However, in 91 patients from the other three trials, ketamine is effective in treating chronic PTSD (risk ratio = −3.66, 95% CI, −7.05 to −0.27; *P* = 0.03).

**Conclusion:**

Ketamine was not effective on lower the PTSD incidence for soldiers on the battlefield, nor on the PTSD-scale scores in early PTSD patients. However, it may improve the PTSD-scale scores for chronic conditions.

**Systematic Review Registration:**

https://www.crd.york.ac.uk/prospero/display_record.php?ID=CRD42021255516, PROSPERO, identifier: CRD42021255516.

## Introduction

Posttraumatic stress disorder (PTSD), defined as a “trauma- and stressor-related disorder,” is a common mental illness (1). The lifetime prevalence of PTSD ranges from 1.3 to 12.2%, and the 1-year prevalence is 0.2–3.8%. The prevalence varies according to psychological and biological factors such as social background, country of residence, endocrine, and genrtic factors (2). PTSD is also associated with suicidal behavior (3), but the authenticity of the relationship remains unclear. Patients with PTSD may have repeated recall of the traumatic experience, avoidance of traumatic situations, hyperarousal, depression, anxiety, substance use disorders, pain, and other symptoms (4). The existing treatments of PTSD include psychotherapy, medication, and innovative interventions such as acupuncture, transcranial magnetic stimulation, and stellate ganglion block (5–8). Howerer, the efficacy of treatments variesdue to the multiplicity and interdependence of biological features (9–14) and complicated pathogenesis, and thus there is a pressing need for research on the pathogenesis and treatment of PTSD.

Ketamine, an N-methyl-D-aspartate (NMDA) receptor antagonist (15, 16), is widely used as a general anesthetic and offers promising perspectives for activating several neurotransmitter pathways in the brain (17–20). Ketamine also has potent analgesic properties (21, 22), anti-inflammatory effects (23), and antidepressant effects (24). In recent years, studies suggest that intravenous ketamine (a single or repeated dose) can quickly reduce the severity of PTSD symptoms in chronic cases [14.6 (25.9), years (SD)] (25, 26). However, the molecular mechanisms involved in the clinical therapeutic effects of ketamine remain unclear and need further exploration.

In the present study, we performed a meta-analysis and systematic review on the use of ketamine and its effects on PTSD.

## Materials and Methods

### Search Strategy and Inclusion Criteria

This meta-analysis and systematic review were registered with PROSPERO (CRD42021255516) and followed the preferred reporting project for systematic reviews and meta-analysis (PRISMA) recommendations (27).

A detailed systematic review of the following databases was performed from inception to 23 May 2021: Cochrane Central Register of Controlled Trials, Embase, PubMed, and Web of Science. The following search terms were used for PubMed: (“ketamine” OR “Ketalar” OR “Calipsol” OR “Kalipsol”) AND (“PTSD” OR “posttraumatic stress disorder” OR “posttraumatic stress symptoms” OR “acute stress disorder”). Randomized controlled trials (RCTs), case-control studies, and cohort studies were included. Both dichotomous and continuous variables were separately included.

### Data Extraction and Quality Assessment

After preliminary screening of the abstract, if found to be suitable, the full text of this article would be further studied. Two authors (Rui and Guofang) independently reviewed all search results based on inclusion and exclusion criteria. The divergent research items were determined by an independent researcher (Kun). All included trials were reviewed by authors to ensure that they met the eligibility criteria. The risk of bias was assessed using Review Manager 5.3. This evaluation included assessment of random sequence generation, allocation concealment, blinding of participants and personnel, blinding of outcome assessment, incomplete outcome data, selective reporting, and other sources of bias. Disagreements in the process were resolved through discussion until a consensus was reached.

The results from RCT, case-control, and cohort studies related to the terms “Ketamine” and “PTSD” were included in this meta-analysis. We excluded correspondences, poster abstracts, texts with no NCT results (National ClinicalTrial.gov), paired comparisons, and manuscripts for which the full text could not be obtained.

Two investigators (Rui and Guofang) independently screened the literature based on the above inclusion and exclusion criteria. By revisiting the original text and performing any appropriate calculations or conversions, any discrepancies found were resolved. Intuitively extracted data included the first author, publication time, participants, study interventions, types of outcomes, reasons for exclusion, study control, and duration of follow-up.

### Data Analysis

The primary outcomes were the effects of ketamine on the occurrence of PTSD in the later stage of the wounded or burn soldiers on the battlefield. The second outcome was the effect of ketamine on patients with diagnosed early PTSD (usually 1–3 months after traumatic events) andchronic (more than 3 months) PTSD based on multiple psychological assessment scales. The Mantel-Haenszel (M-H) method was used to pool dichotomous data and to compute the risk ratio (RR) with its corresponding 95% confidence interval (CI). The inverse variance (IV) method was used to pool continuous data and to calculate the standardized mean difference with a 95% CI. Random effects models were used with or without apparent heterogeneity based on *I*^2^ > 50% compared to the fixed effects model (*I*^2^ < 50%) (28, 29). Sensitivity analysis was performed to assess the stability of the meta-analysis. *P* < 0.05 was considered statistically significant. Review Manager software (RevMan, version 5.3.5. Copenhagen: The Nordic Cochrane Center, The Cochrane Collaboration, 2014) was used for this study.

## Results

### Study Characteristics

**Figure 1:**
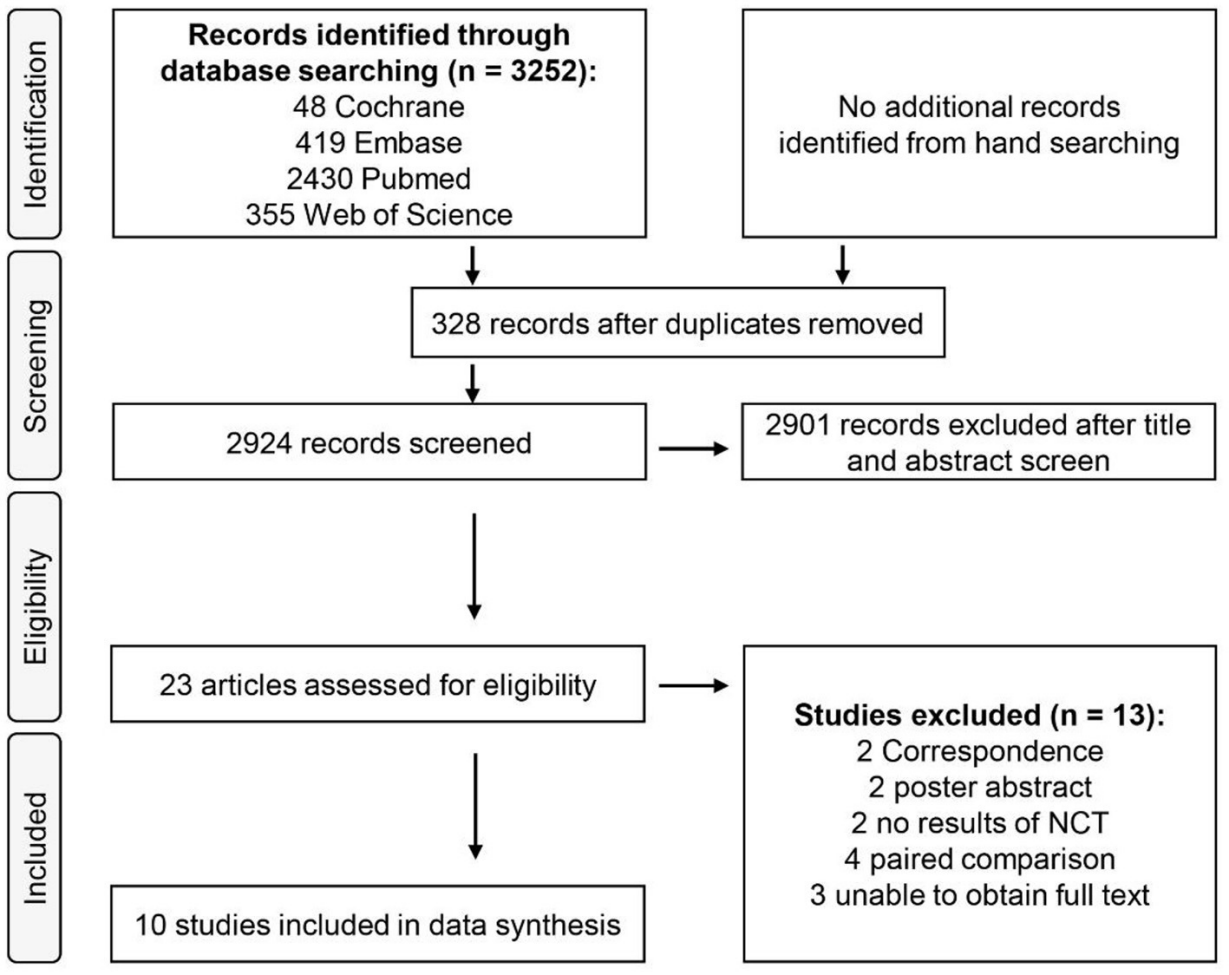
PRISMA flowchart of included and excluded studies.

**Figure 2:**
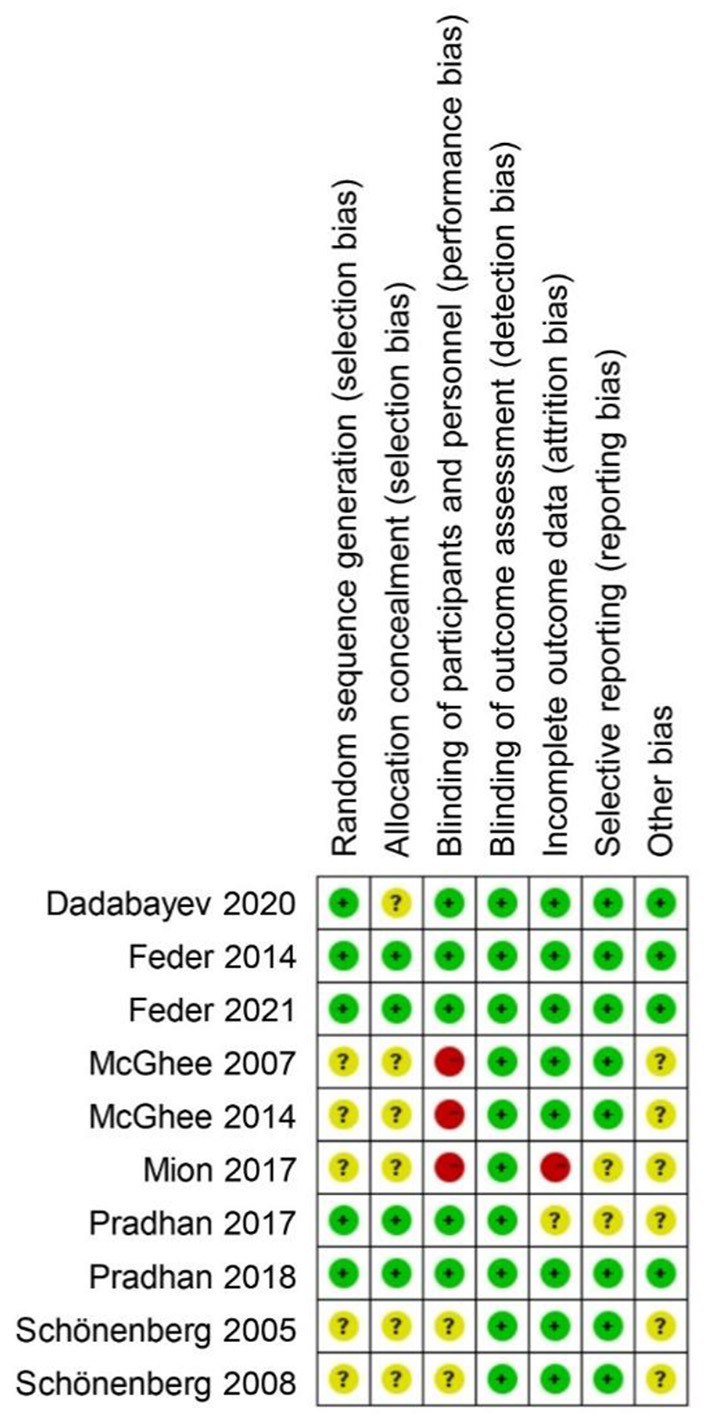
Summary table illustrating risk of bias results for included studies. Green circle, low risk; yellow circle, some concerns; red circle, high risk.

The selection process is summarized in Figure 1. The characteristics of the trials are presented in Table 1. An initial search identified 3,252 articles, including 328 duplicates. Thus, 2,924 articles were screened according to the previously formulated retrieval strategies. After the exclusion of 2,901 articles based on title or abstract, 23 full-text articles were reviewed and assessed for eligibility. Of these, 13 were further excluded because two were correspondences without chart data (38, 39), two were poster abstracts without chart data (40, 41), two were NCTs finished without posted results [NCT02398136, NCT02655692], three were abstracts without available full texts (42–44), and four were paired comparisons, which were not considered in this meta-analysis (Figure 1) (45–48). Finally, 10 studies that met (five RCT studies, three case-control studies, and two cohort studies) our predetermined inclusion criteria were included in this study. The results of the risk of bias assessment are shown in Figure 2. A total of 10 studies reported the effects of ketamine on the occurrence of PTSD after battlefield rescue or in burned patients (31–33) as well as symptom changes in early PTSD patients with a dose of ketamine (0.5 mg/kg) (30, 34, 37) and the same dose for chronic PTSD patients (25, 26, 35).

**Table 1:** Included study characteristics, intervention, duration, scales, and study method.

**Sources**	**Subjects**	**Intervention (*n*, dose)**	**Duration of PTSD**	**Scales**	**Study method**
Dadabayev et al. (30)	CP+PTSD	Ketamine (11, 0.5 mg/kg)	≥3 months (both)	IES-R	RCT
		Ketorolac (10, 15 mg)			
Feder et al. (26)	PTSD	Ketamine (22, 0.5 mg/kg)	Mean ± SD (year)	CAPS-5	RCT
		Midazolam (19, 0.045 mg/kg)	Ketamine: 14.2 ± 12.3	IES-R	
			Midazolam: 11.9 ± 14.0		
Feder et al. (25)	PTSD	Ketamine (15, 0.5 mg/kg)	Mean ± SD (year)	CAPS-5	RCT
		Midazolam (15,0.045 mg/kg)	Ketamine: 15.1 ± 17.8	IES-R	
			Midazolam: 14.6 ± 7.8		
McGhee et al. (31)	PTSD/non-	Ketamine (119, not list)	Not list	PCL-M (Military)	Case-control
		No ketamine (28, not list)			
McGhee et al. (32)	PTSD/non-	Ketamine (189, not list)	Not list	PCL-M (Military)	Case-control
		No ketamine (28, not list)			
Mion et al. (33)	PTSD/non-	Ketamine (41, not list)	Not list	PCL	Case-control
		Midazolam (26, not list)			
Pradhan et al. (34)	PTSD	Ketamine (5, 0.5 mg/kg)	≥ 6 months (both)	CAPS	RCT
		Normal saline (4, not list)		PCL	
Pradhan et al. (35)	PTSD	Ketamine (10, 0.5 mg/kg)	Mean ± SD (year)	CAPS	RCT
		Normal saline (10, not list)	Ketamine: 15.0 ± 9.0	PCL	
			Normal saline: 15.4 ± 11.7		
Schönenberg et al. (36)	PTSD	Opioids (27, not list)	Mean ± SD (month)	PDEQ	Cohort
		Racemic ketamine (17, not list)	Opioids: 14.0 ± 5.3	ASDS	
		(S)-ketamine (12, not list)	Racemic ketamine: 12.8 ± 5.8(S)-ketamine: 10.7 ± 3.4	IES	
Schönenberg et al. (37)	PTSD	Non-Opioids (13, not list)	Early post-traumatic (not list)	PDEQ	Cohort
		Opioids (24, not list)		ASDS	
		Ketamine (13)			

*ASDS, Acute Stress Disorder Scale; CAPS, Clinician-Administered PTSD Scale; CP, chronic pain; IES-R, Impact of Event Scale-Revised; PCL, PTSD Checklist; PDEQ, Peritraumatic Dissociative Experiences Questionnaire; RCT, Randomized Controlled Trial*.

The 10 studies involved a total of 705 patients and were allocated to eligible study groups (Figure 1). Of these, a total of 442 patients were assigned to ketamine or (S)-ketamine, and 263 were assigned to the control (i.e., ketorolac, midazolam, normal saline, and opioid or placebo, Table 1). Patients in five studies (Table 1) (31–35) received a diagnosis of PTSD assessed with a PTSD checklist (PCL) (49). Patients in four studies (Table 1) (25, 26, 34, 35) received the Clinician-Administered PTSD Scale (CAPS) (50). Patients in 4 studies (Table 1) (25, 26, 30, 35) received the Impact of Event Scale-Revised (IES-R) (51). Patients in two studies (Table 1) (36, 37) were assessed based on the Acute Stress Disorder Scale (ASDS) (52).

### Ketamine for the Multivariate Effect of PTSD

**Figure 3:**
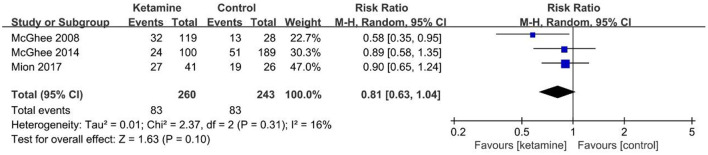
Forest plot of the prevalence of PTSD in battlefield analgesia by ketamine. M–H, Mantel–Haenszel.

Three studies (31–33) including 503 patients (ketamine 349, control 154) in the battlefield reported no significant reduction in PTSD prevalence (Figure 3) with ketamine (no dose list) based on a risk ratio (95% CI) of 0.86 (0.61–1.20), *p* = 0.38, *I*^2^ = 52% compared with the control.

**Figure 4:**
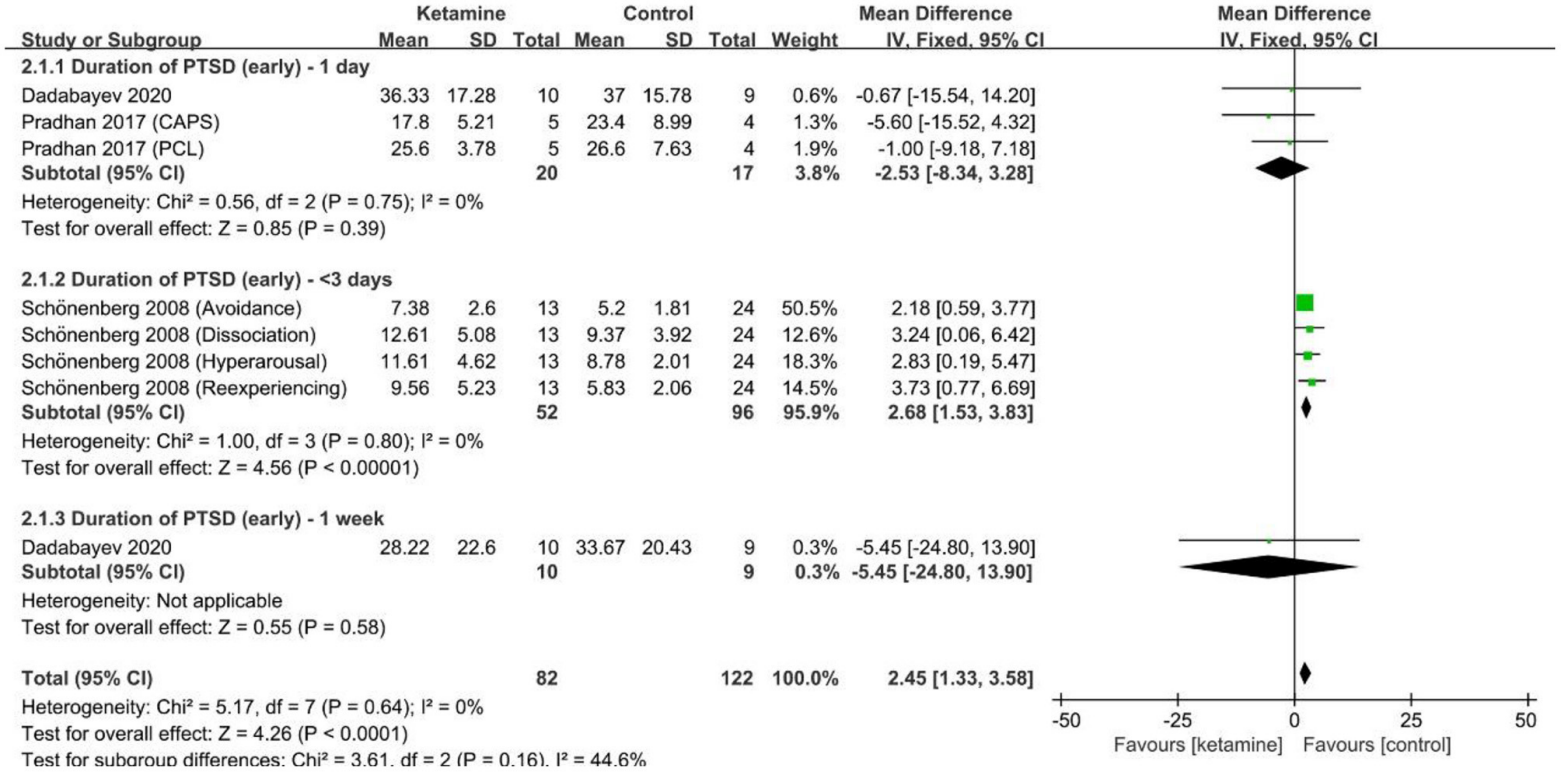
PTSD-scale scores of short duration (months) by ketamine administration (ketamine vs. control). CAPS, Clinician-Administered PTSD Scale; PCL, PTSD Checklist; IV, inverse variance; SD, standard deviation.

Three studies (30, 34, 37) including 67 patients (ketamine 29, control 38) reported scores that changed in early PTSD based on ASDS (four subscales, including dissociation, reexperiencing, avoidance and hyperarousal) (37), IES-R, PCL, and CAPS scales (Figure 4). Taken together, compared with the control, ketamine (0.5 mg/kg) aggravated PTSD symptoms (Figure 4) by a mean difference (95% CI) of 2.45 (1.33–3.58), *p* < 0.001, *I*^2^ = 0%. Subgroup analysis for each time point suggested that 1 day after management (ketamine 16, control 14), ketamine (0.5 mg/kg) showed no significance of aggravation in PTSD symptoms by a mean difference (95% CI) of −2.50 (−8.26 to 3.27), *p* = 0.40, *I*^2^ = 0%; <3 days after management (ketamine 13, control 24), ketamine aggravated PTSD symptoms by a mean difference (95% CI) of 2.68 (1.53–3.83), *p* < 0.001, *I*^2^ = 0%; 1 week after management (ketamine 10, control 9), ketamine (0.5 mg/kg) showed no significance of aggravation in PTSD symptoms by a mean difference (95% CI) of −5.45 (−24.80 to 13.90), *p* = 0.58. Including one study without infusion time [49], ketamine aggravated PTSD symptoms (Supplementary Figure 1) based on a mean difference (95% CI) of 1.72 (0.95–2.48), *p* < 0.001, *I*^2^ = 0%.

**Figure 5:**
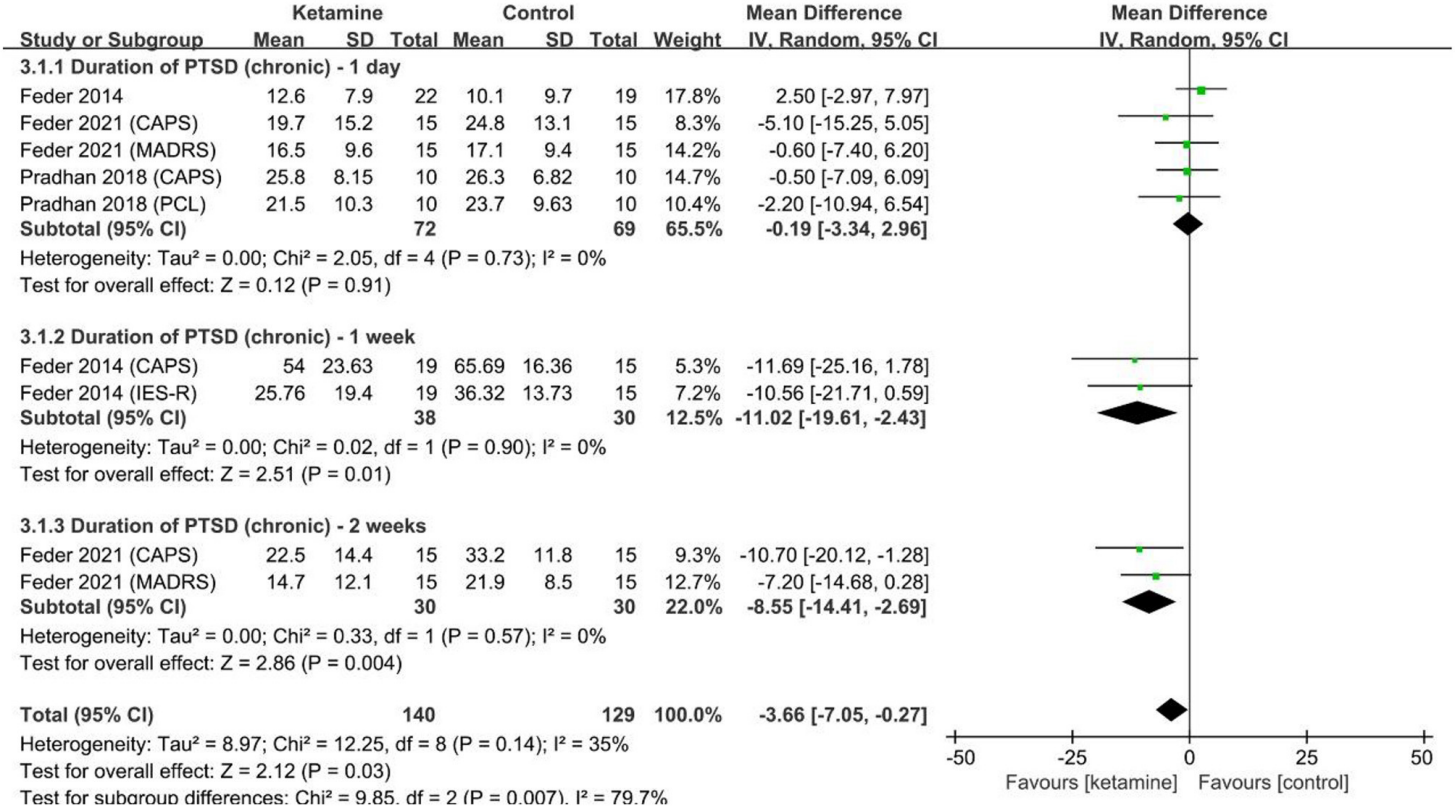
PTSD-scale scores of long duration (chronic, years) patients by ketamine. administration (ketamine vs. control). CAPS, Clinician-Administered PTSD Scale; MADPS, Montgomery-Asberg Depression Rating Scale; IES-R, Impact of Event Scale–Revised score; PCL, PTSD Checklist; IV, inverse variance; SD, standard deviation.

Our third coprimary outcome, including 91 patients (ketamine 47, control 44) (25, 26, 35), reported that scores changed over a long duration of PTSD (years) according to the CAPS, IES-R and PCL scales (Figure 5). Compared with the control, ketamine (0.5 mg/kg) relieved PTSD symptoms (Figure 5) by a mean difference (95% CI) of −3.66 (−7.05 to −0.27), *p* = 0.03, *I*^2^ = 35%. Subgroup analysis for each time point suggested that 1 day after management (ketamine 47, control 44), ketamine (0.5 mg/kg) showed no significance of relief in PTSD symptoms by a mean difference (95% CI) of −0.19 (−3.34 to 2.96), *p* = 0.91, *I*^2^ = 0%; 1 week after management (ketamine 19, control 15), ketamine (0.5 mg/kg) relieved PTSD symptoms by a mean difference (95% CI) of −11.02 (−19.61 to −2.43), *p* = 0.01, *I*^2^ = 0%; 2 weeks after management (ketamine 15, control 15), ketamine (0.5 mg/kg) relieved PTSD symptoms by a mean difference (95% CI) of −8.55 (−14.41 to −2.69), *p* = 0.004, *I*^2^ = 0%.

## Discussion

In the present study, ketamine had no effect on the development of PTSD for injured soldiers (e.g., firearm, explosive, burn, or accident) on battlefields. The symptoms were aggravated in the patients who had been diagnosed with early PTSD after the administration of ketamine. However, the symptoms were ameliorated in patients with chronic PTSDafter ketamine treatment. The results indicate that ketamine treatment was not related to the development of PTSD but was associated with the disease duration in PTSD patients.

It is traditionally believed that ketamine administration is associated with an increased incidence of PTSD. However, in our study, no significant difference was noted between the development of PTSD and ketamine treatment in injured soldiers. Although the explanation for the difference in the results is unclear, our findings are also supported by Mion et al. (33) given that ketamine did not increase the risk of PTSD. The use of ketamine during a stressful event may reduce the preventive effect, but have no effect on the subsequent development of PTSD (31, 33).

To our surprise, we found that ketamine can significantly alleviate the symptoms of chronic PTSD without obvious symptoms of psychosis or mania. This finding has application significance for the clinical therapeutic effects of ketamine. Previous reports by Lapidus et al. (24), who used ketamine for major depressive disorder (MDD), found rapid antidepressant effects of intranasal ketamine on MDD (53). Interestingly, a similar therapeutic effect on PTSD was noted after the administration of ibuprofen, an NSAID (54). Possible mechanisms underlying ketamine's therapeutic effects could be related to ketamine rapidly increasing synaptic connections in the prefrontal cortex and reversing the behavioral and neuronal changes caused by chronic stress in rats, activating mammalian-targeted rapamycin signaling pathways and stimulating brain-derived neurotrophic factor signals (26, 55, 56). In addition, repeated injections of ketamine are safe for patients with chronic PTSD and are generally well-tolerated with only short-term mental and haemodynamic side effects (25).

In contrast, through comprehensive data analysis, we also found that ketamine can aggravate the symptoms for diagnoses of PTSD with a short-term course, including within 1 week. The half-life of ketamine under anesthesia is 2–3 h with psychosimulating symptoms lasting up to 3 days (36), and the effect can be observed up to 1 week after a single dose (Figure 4). One possibility is that ketamine overstimulates the stress-induced glutamate-glucocorticoid interaction in the early stage of trauma, which subsequently leads to stronger dissociation symptoms and fragmentation to consolidate traumatic memories and aggravate the symptoms (37). In addition, ketamine can rapidly induce synapses in the brain-derived neurotrophic factor (BDNF) pathway, increase proinflammatory cytokines, and subsequently activate microglia to aggravate the symptoms of PTSD (57).

We cannot exclude the possibility that the difference in ketamine's effect is caused by different statistical methods and standards. In addition, the results confirmed that ketamine treatment could ameliorate the symptoms caused by chronic PTSD. However, we need to further verify this notion at both the animal research level and in clinical studies. The definite mechanism of the positive or negative effect of ketamine remains unclear.

In summary, this is the first meta-analysis to analyse the relationship between ketamine and the progression of PTSD. In summary, the results from the current study demonstrated that as analgesia proceeds, the development of PTSD will not be affected by the administration of ketamine. The chronicPTSD but not the early PTSD is alleviated by ketamine treatment.

## Data Availability Statement

The original contributions presented in the study are included in the article/Supplementary Material, further inquiries can be directed to the corresponding author/s.

## Author Contributions

RD and GL designed the study. RD, KN, and RH searched articles and analyzed data. JX and ZZ analyzed data. GL searched articles and wrote the manuscript. RH and GL revised the manuscript. YS provided academic advice to the study. All authors contributed to the article and approved the submitted version.

## Funding

This work was supported by grants from National Natural Science Foundation of China (82173241).

## Conflict of Interest

The authors declare that the research was conducted in the absence of any commercial or financial relationships that could be construed as a potential conflict of interest.

## Publisher's Note

All claims expressed in this article are solely those of the authors and do not necessarily represent those of their affiliated organizations, or those of the publisher, the editors and the reviewers. Any product that may be evaluated in this article, or claim that may be made by its manufacturer, is not guaranteed or endorsed by the publisher.
