# Supplementary material for: The Multivariate Effect of Ketamine on PTSD: Systematic Review and Meta-Analysis
Source: Front Psychiatry. 2022 Mar 9;13:813103. doi: 10.3389/fpsyt.2022.813103 (PMC8959757; doi:10.3389/fpsyt.2022.813103)
Supplement: Supplementary file 1 [file Data_Sheet_1.docx]

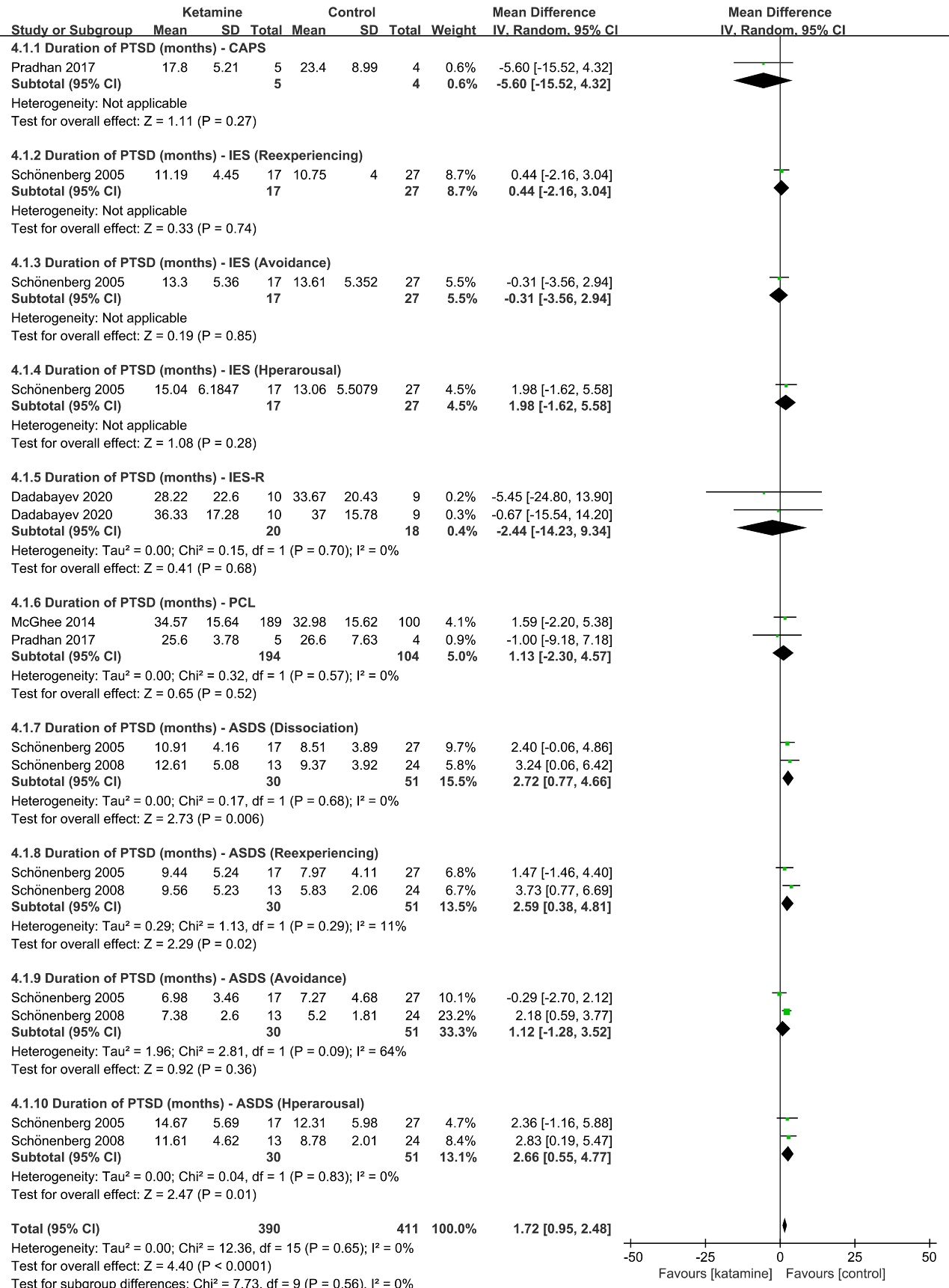


Fig. S1 PTSD-scale scores of short duration (months) by ketamine administration. (ketamine vs. control, full scales). IV, inverse variance; SD, standard deviation.
